# Supplementary figures and images for: Investigation of the critical factors required to improve the disclosure and discussion of harm with affected women and families: a study protocol for a qualitative, realist study in NHS maternity services (the DISCERN study)
Source: BMJ Open. 2022 Feb 3;12(2):e048285. doi: 10.1136/bmjopen-2020-048285 (PMC8814750; doi:10.1136/bmjopen-2020-048285)

## Appendix 2:

## Study Recruitment And Data Management For Study Phase 1b

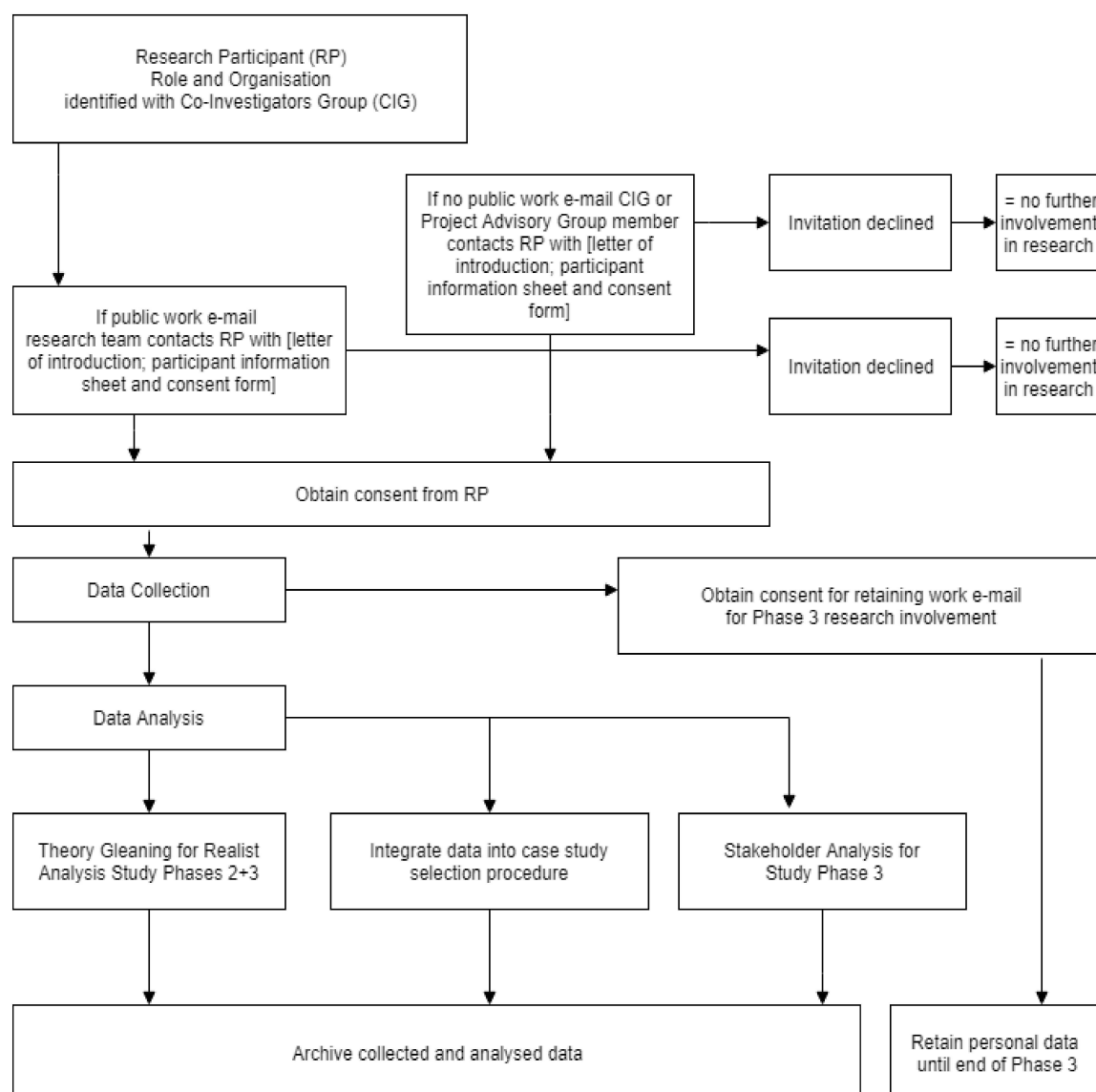

Supplement: Supplementary data [file bmjopen-2020-048285supp002.pdf]
