# Supplementary material for: Investigation of the critical factors required to improve the disclosure and discussion of harm with affected women and families: a study protocol for a qualitative, realist study in NHS maternity services (the DISCERN study)
Source: BMJ Open. 2022 Feb 3;12(2):e048285. doi: 10.1136/bmjopen-2020-048285 (PMC8814750; doi:10.1136/bmjopen-2020-048285)
Supplement: Supplementary data [file bmjopen-2020-048285supp003.pdf]

APPENDIX 3: STRATEGY FOR SHORT-LISTING POTENTIAL CASE STUDY SITES

4 potential study sites have been identified using a staged sampling frame to identify a purposive sample of organisations or services that are undertaking improvement work in OD in NHS maternity care. We have considered maternity networks, trusts or service areas or services as potential study sites. The staged sampling frame has been two-staged:
Stage 1:
Long listing potential study sites from public data sets (notably trust-level scores on 'Transparency: Learning from Mistakes League' and the NHS staff survey). This data is reported annually and shows variations between all NHS acute trusts (from outstanding levels of openness and transparency [level 1] to poor reporting culture [level 4]. This data set is established from selected metrics from the annual NHS staff survey and from the National Reporting and Learning System. Service-level scores from the annual NHS staff survey, '*Reporting on 'Errors and Incidents', selected items* [KF 29; 30;31] (see Box 1).

Box 1: 'Errors and Incidents': items from the annual NHS staff survey (by trust and location)

**Survey Item**	**Error and Incidents: by Trust and by Trust Location (Women's Health)**
KF 29	staff reported errors, near misses or incidents witnessed in last month (percentage score)
KF 30	staff reported fairness and effectiveness of procedures for reporting errors, near misses and incidents (scale summary score)
KF 31	staff reported confidence and security in reporting unsafe clinical practice (scale summary score)

These key findings are benchmarked against others trusts and for different locations [or divisions] within that trust. Women's Health, Gynaecology and Obstetrics, as well as Midwifery are all locations. Therefore, we have been able to establish a comparative picture of how staff experience incident investigation, across and between our listed sites in these areas and listed acute trusts.

We have long-listed those trust and service areas where there is good or improving performance reported in fields that indicate candour (for example, transparency, incident reporting, staff reported organisational or service responsiveness of incident reporting).
Stage 2:
Short-listing long-listed sites using combined soft intelligence from national and regional stakeholders (at Phase 1 scoping study).

Due to the sensitivity of the research topic for frontline staff as well as for services and trusts, and the anticipated challenges of organisational engagement in some trusts, services and networks, our sampling frame follows Stake's (2013) principles to:

i. maximise chances of ongoing engagement of participants in the research
ii. provide diversity of cases across contexts
iii. ensure that all cases provide good opportunities to learn about complexity and context

Therefore, we shortlisted only those services, trusts and networks identified as potentially available as case study sites by stakeholder or PAG recommendation.

Reference:

Stake R. E. 2013. *Multiple Case Study Analysis*. Guildford Press.
