# Supplementary material for: Investigation of the critical factors required to improve the disclosure and discussion of harm with affected women and families: a study protocol for a qualitative, realist study in NHS maternity services (the DISCERN study)
Source: BMJ Open. 2022 Feb 3;12(2):e048285. doi: 10.1136/bmjopen-2020-048285 (PMC8814750; doi:10.1136/bmjopen-2020-048285)
Supplement: Supplementary data [file bmjopen-2020-048285supp004.pdf]

Appendix 4: Recruitment strategy for organisations and Individuals.

Sample Identification for Study Phase 2

Study Phase 2 (up to 4 case studies, with each case study comprising focused (group or individual staff) non-participant observations (n=25 hours); in-depth interviews with NHS staff and patients (approximately n=12); and collection and analysis of redacted documentary data (n=20 hours). Some return interviews may be arranged with staff who lead the OD improvement work (n=approximately 3 per site).

Recruitment of case study sites into the study will involve a range of approaches in order to maximise opportunities of team, service, trust and/or network engagement. These are:

a. <u>sampling strategy for the potential case study sites</u>:
The identification of any site for potential recruitment will be on the basis of their already being active in maternity quality and safety improvement work and on the basis of their likely interest in engaging with the study team (as identified by Phase 1 interviewees and our independent project advisors). We will therefore ensure that prior to our sampling for diversity or context, we have sampled to maximise our chances of ongoing engagement of services, trusts and/or networks in the study. In addition, we will identify potential sites by internet searches. These searches will assist in our closer identification of maternity safety and learning ‘candour’ collaborative or working groups (for example, as developed in the Health Innovation Networks or as supported by local NHS staff or parent/maternity support groups).

b. <u>use of a recommended framework for enhancing study participant recruitment:</u>
We understand that the recruitment of organisations and networks, as well as of those who work within them, is a multi-layered process. We will draw on McDonald’s (2011) adapted business-model approach to recruitment to inform our recruitment plan.

We will undertake:

i. **Early and widespread familiarisation of the research project (the study question, along with the ongoing promotion of study value, legitimacy and potential benefits) across NHS maternity services.** We will initiate promotion of the study (and of the senior researchers involved) from study start date. This promotion will be established and sustained through social media (Twitter; a project blog and, on the advice of our independent project advisors, a study update newsletter) until project completion. Prior to Phase 1, and after the study has been contracted, our study team and independent project advisors will be encouraged to ‘spread the word’ on the research through regional and national networks. This familiarisation and ongoing promotion will be underpinned by a simple message of ‘what the study stands for’ and how it can be recognised.

ii. **Early identification of potential case study sites.** We will be anticipating potential sites throughout the Phase 1 research. This will include establishing short ‘case study’ profiles to aid us with sampling following Stake’s (2013) case study sampling principles as well as by making early contact with potential sites through gatekeepers identifiable on public websites. These initial contracts (and a response after one email contact and one email reminder) will aid our assessment of their interest in potential participation.

iii. **Anticipation of multiple dimensions of recruitment**. We are aware that access to services, trusts and/or networks will require successful engagement with multiple sponsors and gatekeepers, both formally and informally. We will use tailored

messages to engage different interest groups (for example, the potential benefits of involvement in the study for patient representatives will differ from those for a group of junior clinicians or managers). We will also identify the range of influential sponsors, champions and change agents within potential case study sites to enlist their ongoing support.

iv. **Assurance to potential participants (organisations and individuals) of the scope and limits of the research across and within case study sites.** Macdonald (2011) highlights the necessity of clarifying the expectations of organisational participation and of the scope and limits of the research to be undertaken to all gatekeepers. This will be particularly important for the case-studies as participants or gatekeepers will be concerned that they may have less control over information shared through survey or interview methods. We will reiterate in all communication materials, that our non-participant observations are focused on the contexts and routines that make OD possible and that we will not be expecting to observe actual OD meetings with women or families (see case-study methods described below). We will not be recruiting women or families from the case study sites as study participants.

v. **Emphasis on the actual and potential benefits of research participation to key gatekeepers as well as to individual study participants**. We are assuming that there will be some reluctance of some potential participants to engage with this study topic. However, we have sought to enhance to benefits to participants by our study design (Phase 3 offers opportunities for networking, social interaction and sharing experiences both within the sites, from our Women and Family Forums and from our Summative Interpretive Forum). Our communications will highlight these opportunities.

vi. **The engagement of staff as potential and actual research participants will be facilitated by short presentations of the study and key study messages by the researchers at local unit meetings in different service areas**. By identified quality and safety improvement champions and by snowballing through clinical or project teams. The ongoing development of ongoing research relationships, with two named and experienced health service ethnographers, will further support recruitment processes.
