# Supplementary material for: Investigation of the critical factors required to improve the disclosure and discussion of harm with affected women and families: a study protocol for a qualitative, realist study in NHS maternity services (the DISCERN study)
Source: BMJ Open. 2022 Feb 3;12(2):e048285. doi: 10.1136/bmjopen-2020-048285 (PMC8814750; doi:10.1136/bmjopen-2020-048285)
Supplement: Author's manuscript [file bmjopen-2020-048285.draft_revisions.pdf]

BMJ Open

BMJ Open is committed to open peer review. As part of this commitment we make the peer review history of every article we publish publicly available.

When an article is published we post the peer reviewers' comments and the authors' responses online. We also post the versions of the paper that were used during peer review. These are the versions that the peer review comments apply to.

The versions of the paper that follow are the versions that were submitted during the peer review process. They are not the versions of record or the final published versions. They should not be cited or distributed as the published version of this manuscript.

BMJ Open is an open access journal and the full, final, typeset and author-corrected version of record of the manuscript is available on our site with no access controls, subscription charges or pay-per-view fees (http://bmjopen.bmj.com).

If you have any questions on BMJ Open's open peer review process please email info.bmjopen@bmj.com

BMJ Open

INVESTIGATION OF THE CRITICAL FACTORS REQUIRED TO IMPROVE THE DISCLOSURE AND DISCUSSION OF HARM WITH AFFECTED WOMEN AND FAMILIES: STUDY PROTOCOL FOR A QUALITATIVE, REALIST STUDY IN NHS MATERNITY SERVICES [THE DISCERN STUDY].

Journal:	*BMJ Open*
Manuscript ID	bmjopen-2020-048285
Article Type:	Protocol
Date Submitted by the Author:	01-Jun-2021

Complete List of Authors:	Adams, Mary; King's College London, Department of Women's and Children's Health, Faculty of Life Science and Medicine, King's College, London. 10th floor. North Wing, St. Thomas' Hospital. Westminster Bridge Road, London SE1 7HE<br>Iedema, Rick; King's College London, Centre for Team-Based Practice and Learning in Health Care, Guy's Campus. London SE1 1UL<br>Heazell, Alexander; University of Manchester, Maternal and Fetal Health Research Centre<br>Treadwell, Maureen; Birth Trauma Association<br>Booker, Maria; Birthrights<br>Bevan, Charlotte; Sands, stillbirth and neonatal death society<br>Hartley, Julie; King's College London, Department of Women's and Children's Health, Faculty of Life Science and Medicine, King's College, London. 10th floor. North Wing, St. Thomas' Hospital. Westminster Bridge Road, London SE1 7HE<br>Sandall, Jane; Kings College, London, Department of Women's and Children's Health, Faculty of Life Science and Medicine, King's College, London. 10th floor. North Wing, St. Thomas' Hospital. Westminster Bridge Road, London SE1 7HE

Keywords:	Quality in health care < HEALTH SERVICES ADMINISTRATION & MANAGEMENT, Organisation of health services < HEALTH SERVICES ADMINISTRATION & MANAGEMENT, Maternal medicine < OBSTETRICS, Protocols & guidelines < HEALTH SERVICES ADMINISTRATION & MANAGEMENT

SCHOLARONE™
Manuscripts

INVESTIGATION OF THE CRITICAL FACTORS REQUIRED TO IMPROVE THE DISCLOSURE AND DISCUSSION OF HARM WITH AFFECTED WOMEN AND FAMILIES: STUDY PROTOCOL FOR A QUALITATIVE, REALIST STUDY IN NHS MATERNITY SERVICES [THE DISCERN STUDY].

Dr Mary Adams, Professor Rick Iedema, Dr Alexander Heazell, Ms Maureen Treadwell, Ms Maria Booker, Ms Charlotte Bevan, Dr Julie Hartley and Professor Jane Sandall

Corresponding Author:
Dr Mary Adams
Department of Women and Children's Health
Faculty of Life Science and Medicine, King's College London
5th Floor, Becket House
1 Lambeth Palace Road
London SE1 7EU

mary.adams@kcl.ac.uk
Tele: 07717473227

Co-Author Details:
Professor Rick Iedema, King's College, London. England.
Professor Alexander Heazell, University of Manchester. England.
Ms. Maureen Treadwell, Birth Trauma Association. England.
Ms. Maria Booker, Birthrights. England.
Ms. Charlotte Bevan, Stillbirth and Neonatal Death Charity. England
Dr. Julie Hartley, King's College, London. England.
Professor Jane Sandall, King's College, London. England.

Key Words
Truth Disclosure
Professional-Patient Relations
Patient Safety
Error Disclosure
Obstetric Departments

Word Count: 3,744.

For peer review only

INVESTIGATION OF THE CRITICAL FACTORS REQUIRED TO IMPROVE THE DISCLOSURE AND DISCUSSION OF HARM WITH AFFECTED WOMEN AND FAMILIES: STUDY PROTOCOL FOR A QUALITATIVE, REALIST STUDY IN NHS MATERNITY SERVICES [THE DISCERN STUDY].

ABSTRACT

INTRODUCTION
Patients and families are entitled to the disclosure and discussion of healthcare incidents affecting them. This can reduce everyone's distress and contribute to learning and safety improvement. Complex barriers prevent effective disclosure and continue in the English NHS, despite a legal duty of candour. NHS maternity services are the focus of significant efforts to improve candour with women and families after safety incidents. There is limited understanding of how maternity providers can achieve this, and the consequences for different groups involved.

METHODS and ANALYSIS
A 27-month, three-phased realist evaluation study to identify the critical factors and situations that contribute to improvements in the disclosure and discussion of incidents with affected women/families. The evaluation asks 'what works, for whom, in what circumstances, in why respects and why?'.
Phase 1 to establish working hypotheses of the key factors and outcomes of interventions to improve disclosure and discussion, by realist literature review and in-depth one-to-one realist interviews with key stakeholders [n=approximately 20]
Phase 2 to refine or overturn hypotheses, by in-depth ethnographic case-study analysis using triangulated qualitative methods (non-participant observation; return interviews [n=12] and documentary analysis) in each of up to 4 purposively sampled NHS trusts.
Phase 3 to refine hypotheses and design outputs during 7 interpretive forums.

ETHICS AND DISSEMINATION: University Ethical Approval has been acquired for Phase 1. National Research Ethical Approval has been acquired for Phases 2 and 3. Briefing documents and tailored summaries for managers; actionable guidance for clinical teams and for women/families (written and video); lay summaries of study findings; in-depth analysis of improvement processes; academic papers and HS&DR report. Tailored dissemination to maximise academic and societal impact. The views and interests of different women/family groups is represented throughout all study stages.

STRENGTHS AND LIMITATIONS OF THIS STUDY

- This is the first study to investigate the contingencies and consequences of the implementation of interventions supporting the disclosure and discussion of harm with women/families following clinical incidents in the NHS maternity care.
- Project methodology developed for this study includes co-design (between social scientists, clinicians and charity/association and user representatives) of study questions, research focus, study outputs and dissemination strategy. This approach has wider applicability in health service research studies.
- Adopting a realist approach, the study identifies 'key ingredients for potential success' for maternity providers already engaged in the interventions and improvements.
- The study does not set out to examine reasons for disinterest or disengagement among some maternity providers.

Impact of COVID-19 on Protocol
- Delays in progressing Phase 2 due to COVID-19 related delays in acquiring local provider and National Research Ethics Approval.
- Revision of study methods: (site visits and interviews) to be conducted 'on-line' until further notice.

For peer review only - http://bmjopen.bmj.com/site/about/guidelines.xhtml

Introduction

Besides patients' entitlement to the disclosure of clinical incidents affecting them[i], when disclosure processes are conducted well, they can reduce the emotional harm to all involved[ii], provide valuable opportunities for learning for improvement[iii,iv] and may reduce public or private expenditures on investigatory processes and legal and financial claims[v]. In the longer-term, disclosure practices may revise patient-clinician hierarchies and/or encourage public trust in healthcare[vi].

However, there is often disagreement or uncertainty over the principles, purpose and impact of disclosure practices. Their enactment often falls short of the expectations of harmed individuals[vii], professional or organisational guidelines[viii], and regulatory directives[ix]. In the UK, fears of professional investigation, loss of reputation, as well as blaming behaviours, are common deterrents to more open communication with patients. Inflexible organisational procedures and limited investment in the training and support of clinicians undertaking disclosure and discussions with patients can mean that the needs of patients are not properly heard or responded to[x].

In the English NHS, each clinician's professional duty of honesty[xi], co-exists with 'Being Open' policy guidance[xii] that is now underpinned by a statutory duty of candour on health providers[xiii]. In NHS maternity services, an array of national and local safety improvement programmes is ongoing to reduce safety incidents and to improve the care of women/families when serious incidents happen. Since 2017, in England, there have been further efforts to improve incident investigation and women/family involvement in these processes[xiv,xv]. One driver for these interventions is the reduction of the escalating costs of clinical negligence claims in NHS maternity care[xvi].

There is limited understanding of how, in what situations, to what effect and for whom these various improvement programmes are being implemented in maternity services and service areas, and their consequence for women/families, clinicians and organisations. Evidence is required for understanding which improvements address the needs and interests of these different groups when a harmful incident happens and how.

Methods and Analysis

Aims

The primary aim is the development of actionable evidence to inform maternity providers of those factors that, in particular situations, will improve the disclosure and discussion of adverse incidents in NHS maternity care with affected women/families. The secondary aim is co-production of outputs (support materials for women/families; clinicians and managers; practical recommendations for organisations) to benefit these groups during these events.

Question

The overarching research question is 'what are the critical factors that can improve the incidence and quality of Open Disclosure in NHS maternity services?'. We will explore the actual or anticipated consequences of ongoing improvement programmes for different participants in particular contexts.

Patient and Public Involvement and Engagement [PPIE]

The study is co-designed and co-produced with three national PPIE partners, charities and associations representing women/families who have experienced harm in maternity care. These partners are core members of our Co-Investigator Group [CIG]. The work is also informed by a wider Project Advisory Group [PAG] that includes 8 individual woman and families with direct experience of harm in maternity care. The PAG advises on study progress, outputs and dissemination.

Study Design

The ongoing interventions in NHS maternity care comprise policy-led, complex interventions promoted in services in the expectation that incidence and/or quality of women/family involvement in the disclosure and discussion of adverse events will be improved. A realist, theory-driven evaluation approach informs study design. Realism, in health service evaluations, examines the interaction between a complex social intervention or 'programme', the contexts of strategic and everyday implementation work, and the un/intended outcomes for different participants [xvii]. Realist evaluation asks, 'what works, for whom, in what circumstances, in what respects and why?'[xviii]. Therefore, the approach does not seek to establish the overall effect of an intervention, but to identify the critical factors (in realist terms, the 'generative mechanisms') that are likely to produce expected and unexpected effects for different groups in particular circumstances[xix]. The approach enables the analysis of the non-linear, contingent qualities of interventions, including the situated conditions of success and failure.

Realist evaluation follows a staged approach that moves from the identification of initial working and interconnected hypotheses from secondary sources to the 'testing' (refinement or overturning) of these hypotheses using primary data to identify those contingencies that drive and direct improvement[xx].

The 3-phased, 27-month, qualitative study will:

1. establish, by literature scoping and expert stakeholder consultation, a series of working hypotheses (Study Phase 1a).
2. 'test' these working hypotheses by in-depth interviews with a range of national stakeholders (Study Phase 1b) and by ethnographic research (Study Phase 2).
3. agree with national stakeholders and different interest groups and experts, the critical factors required to trigger and sustain interventions to improve the disclosure and discussion of harm with women/families in NHS maternity care; co-design outputs to support these generative factors (Study Phase 3).

See Appendix 1 for the DISCERN study flow diagram.

Methods for Study Phases

Phase 1a

For peer review only - http://bmjopen.bmj.com/site/about/guidelines.xhtml

For peer review only

The PROSPERO registered realist review will identify and extract literature from social science, nursing and medical and health service management electronic databases. The purpose of the review is descriptive and will identify papers and documents of relevance to disclosure and discussion of harm in international maternity services. Initial scoping searches will identify keywords, synonyms, spelling variations, searching will include both free text and database-specific subject headings (such as MeSH terms); advanced Boolean truncation; 'explode' and other techniques. The database search will be supplemented by 'snowball' strategies, scanning of reference lists from relevant papers and advice from subject experts. Following realist methodology, no specific pre-determined inclusion and exclusion criteria will be used to identify inclusions, however, we will report on areas of study design and evidence weakness and rank inclusions, following RAMSES project guidance. Grey literature, including policy reports, service guidance and public and professional commentary will be retrieved. The search is limited to English language documents. Abstracts of all identified papers will be screened for relevance and rigour. If over 50 papers are identified those most relevant to study aims and objectives will be selected. A structured realist data extraction form will be developed to document item; context; intervention details/indications of mechanism/outcome relationships; factors affecting implementation; evidence of improvement program outcomes.

Thematic analysis of extracted data to develop early hypotheses of critical factors that influence improvements and their consequences for women/families, clinicians and services. One-to-one discussion in the CIG, and consultation with the PAG, comprising academics, policy-makers, patient and public advocates and front-line clinicians, to validate secondary data analysis.

Phase 1b

One-to-one, open-ended, audio-recorded telephone interviews with approximately 20 national stakeholders in service improvement and patient/family engagement in NHS maternity care using a topic guide developed from Phase 1a findings and piloted with co-investigators.

This interview study will (i) explore interviewees' various experiences and views in relation to initial hypotheses[xxi]; (2) gather 'soft intelligence 'on site selection for Study Phase 2; (3) establish stakeholders' views on how to maximise research impact[xxii] for Study Phase 3.

Interviewees will be sampled for maximum diversity across national stakeholder groups. Advice from the CIG and the PAG, along with snowball sampling with interviewees, will guide interviewee approach and selection.

See Appendix 2 for the flow diagram of study recruitment and data management for Study Phase 1b.

Phase 2

Up to 4 NHS maternity services, identified as high-performing or improving (in terms of supporting interventions for strengthening disclosure practices with women/families after harm in maternity care) will be selected as ethnographic case studies. An ethnographic methodology will enable in-depth interpretation of the context and practices of clinical or organisational relationships, including the situated and often taken-for-granted qualities of these practices, including their negotiation and legitimation[xxiii]. This approach is important for understanding the local reception, adjustments and viability of new interventions[xxiv]. Ethnographic case-studies enable detailed data to be analysed dynamically, with respect to particular organisational and service contexts, events and activities, and as a series of comparative case studies.

A case-study comprises 10 x 3-day visits, completed over ten months, with periods between visits for researchers to discuss and compare emergent findings. Initial hypotheses and their refinements or revisions (developed in Study Phase 1) will focus the comparative case-studies. However, they will also document (i) how relevant policy and procedures are promoted, managed and monitored as well as interpreted and evaluated by recipients (along with the challenges and opportunities to this);(ii) factors that influence the 'when and how' of procedural enactments in actual clinical settings by different participants including women/families, clinicians, administrators and management and governance teams; (iii) the interplay of formal and informal local workplace cultures and wider public and policy debates; (iv) personal, team and service histories if disclosure experiences, including their potential to influence wider revisions in the 'Being Open' agenda, patient-clinician-manager relationships and their acceptance as routine.

Our sampling follows social science principles. Due to expected challenges in engaging managers or clinicians in some maternity services, the sampling frame includes only cases of positive deviance in order to maximise engagement in the study. A positive deviance approach holds that behaviours (and situations) the produce poor practice or experience are variations on the same processes that produce successful practices. Therefore, focusing on success is a more effective tactic for understanding how improvements are achieved[xxv]. Sampling includes maximum diversity between services so that opportunities to learning about in-case and cross-case variation is maximised[xxvi] Short-listing of potential case study sites will use public data set scores; information on service provision (size, population served, neonatal care provision).

Further information on sampling framework is described in Appendix 3.

Approach and recruitment of selected organisations, site Principal Investigators [PIs], and key gate-keepers, is tailored from a theory-based, business-model approach to recruitment to clinical studies.

A description of the application of this business model to the study is described in Appendix 4.

Each ethnographic case study will triangulate findings collected by the following methods:

For peer review only - http://bmjopen.bmj.com/site/about/guidelines.xhtml

a. *Focused Observations* [estimated 25 hours] of formal and informal events and routines intended to secure improvement in disclosure of adverse incidents with women/families (e.g. service-level and Trust-level meetings on serious incident reporting and analysis; training activities; promotion and publicity work; and meetings between patient liaison and clinical teams. Observations may include meetings between family members and organisational representatives.
b. *In-depth Return Interviews* with about 12 staff [some at two time-points] involved with, or affected by, the improvement work (e.g. clinical and governance leads; patient support personnel; more junior clinicians).
c. *Documentary Data* including redacted incident reports; meeting minutes, action plans; organisational and service bulletins; training reports; quality and safety improvement reports; and incident reports from families to construct a retrospective picture of OD work within that case study.

With participants' informed consent, in-depth interviews will be audio-recorded on encrypted devices and transcribed verbatim. The observational data will be recorded as low inference notes and later word-processed by that researcher. All documentary data will be redacted before inclusion in the study.

Phase 3

Following initial analysis of case-study data, eight forums of purposively-selected individuals will contribute to the interpretation and validation of findings[xxvii] and co-design, with the CIG, training materials from the study findings. Audio-recorded forum discussions will be thematically analysed to (a) further validate and develop findings on the critical factors and situations that support disclosure processes; (b) support output co-development.

Potential participants will be identified during participation with Phase 1 or 2 and sampled for maximum variation in their experiences of disclosure practice improvements. A maximum of 25 participants will be invited to any one of the 7 local forums. A final, national forum, to include local forum representatives along with national policymakers, will accommodate up to 60 participants and will be externally facilitated. The national event will be hosted at the sponsoring university. Wider consultations of study findings and output development will be with established on-line forums for harmed patients/families and clinicians interested in maternity safety improvement.

Data Analysis

Data will be analysed iteratively, with reading and re-reading of transcripts in refine or overturn initial hypotheses[xxviii]. Some data analysis will be conducted at the close of each Study Phase, to maintain a staged approach to hypotheses refinements. Following familiarisation with the anonymised transcripts, two experienced researchers will independently identify and explore emergent themes in relation to our initial hypotheses of the critical factors that support OD improvements, to what effect and for whom (as identified in Phase 1a). Phase 2 data will also be analysed in a triangulating fashion to develop concrete descriptions of the different factors, contexts and contingencies that foster and shape OD interventions and their various outcomes and effects in particular services. In-case analysis and cross-case comparisons, will help to refine our understanding of which critical factors may be fostered or sustained in different contexts and contingencies and produce different effects and outcomes[xxix]. We will seek to identify convergent and contradictory evidence within and then across the cases. Coding and data management will be organised and audited using qualitative data analysis software, Nvivo (V12). Two more senior researchers will check and help to refine data analysis.

Where possible, cross-tabulations and scattergrams will be used to plot the relationship between apparently anomalous aspects of improvement work in this area and other social variables (e.g. distinctive service or organisational histories, resourcing agreements, or public involvement structures and relationships).

A stakeholder analysis using the RAPID AIIM matrix[xxx] may be used to assist in mapping participant interests in the subject area and their influence over local and national improvement work.

The hypotheses generated will also be developed using conceptual frameworks drawn from the social sciences (for example, Normalisation Process Theory, an Institutional Logics approach, approaches to trust in health care organisations; and perspectives on team and organisational safety and resilience.

Outputs and Dissemination

Outputs will be robust and actionable evidence about the organisational strategies, and team, service and network practices that can support and enhance the quality and extent of OD within NHS maternity care and wider NHS care provision within particular circumstances and with respect to different interest groups. Our findings are intended to impact on three organisational areas of health care: the micro-level of disclosure processes and relationships; meso-level of service management and organisation of these processes; and macro-level of policy. Therefore, our dissemination strategy, with outputs tailored to the audience[xxxi], includes:

- Co-designed briefing documents and guidance for maternity service managers and policy-makers.
- Co-designed information materials (likely, short guidance sheets and video animations for services, clinicians and women/families (and promotion of these by our Stage 3 study participants).
- Accessible public information and lay summaries of study findings disseminated through our dedicated Twitter account and website.
- Peer-reviewed academic journal articles (including the final NIHR study report)
- A summary of findings sent to all study participants who have consented to receive this information.

More widely, the dissemination plan is underpinned by all RCUK identified factors that help to generate academic and societal impact. The research will enhance the evidence base for improving the use and quality of OD in NHS maternity care, influencing policy and practice; produce theoretically informed knowledge of the critical factors for fostering, enhancing and sustaining OD; and will support the growth of networks and knowledge exchange between NHS maternity providers, women/family interest groups and associations, OD policy-makers and non-maternity NHS providers.

For peer review only - http://bmjopen.bmj.com/site/about/guidelines.xhtml

Ethical Considerations

We have identified this study as a high risk, qualitative research study because we are:

- conducting research on a highly sensitive and potentially emotive topic for all concerned.
- interviewing and observing clinicians and managers whose care might have resulted in harm.
- observing meetings with women/families and managers that may become adversarial.

The primary ethical issues requiring mitigation are:

- *non-consent or withdrawal of consent to study participation* (for example, by some staff during research observation of staff meetings and routines). In these situations, we will always respect withdrawal of study participation and we will state this in all participant information and consent forms. We also will seek to focus observations of collective routines on more public workplace activities (e.g. training events)
- *disclosure of negligence* (for example, during staff and during women/family representative interviews). We will make it clear in all participant observation that we have a duty to disclose the negligence of patients. We will establish a system for researchers concerned about negligence to alert the PI within an established time frame and for the prompt notification of the relevant Freedom to Speak Up Guardian.
- *participant engagement* (by trusts, services, units and teams who feel judged by the study sampling procedures and the study topic). We are aware that obtaining initial and ongoing engagement with participants is possible if it is done sensitively and ethically. We will reassure all trusts and services that we will not be approaching, interviewing or observing women and families directly.

d. *Non-malfeasance regarding study participants.* In research information and methods, we will follow current guidance on interviewing people about potentially sensitive topics or traumatic experience[xxxii],[xxxiii] and specialist (one-to-one) advice from our PAG.

e. *Non-malfeasance regarding PPIE representatives* (study co-applicants; PAG members; forum participants). We have structured PPIE representation across the study so that leadership and support roles are clearly defined. We have ensured that all PPIE co-applicants and PAG members are highly experienced in research and engaging with diverse interest groups. We have also ensured linkage between the PPIE PAG lead and the study PAG lead to ensure consistency of support as well as of representation.

Issues of anonymity, confidentiality and informed consent will be addressed in the recruitment of all participants, data collection processes and data storage. The principles of beneficence and non-malfeasance will be adhered to. Throughout the study the MRC/HRA guidance 'GDPR and Data Protection Act 2018: key facts for researchers' has been followed.

Public and Patient Participation and Engagement

To ensure that the views and interests of women/families, and the wider public, remain the focus of the study, our PPI strategy supports representation at all study phases (study design, management, data interpretation, co-development of outputs, reporting of findings and dissemination of research findings, along with other outputs). We have followed the new national standards[xxxiv]. that provide a series of indicators to support structures and behaviours that support PPI in study governance and study impact. The approach reflects current policy emphasis on participatory approaches to healthcare service quality and safety improvement. This is to be achieved by:

(a) The involvement of women/families as well as their charity and association representatives in all stages of the research cycle (study application, research planning, research activity, study steering, write-up and dissemination of findings).
(b) a multi-disciplinary and multi-sector research team that includes public members.
(c) ongoing collaboration with an extensive and varied PAG, including their involvement in the summative forum.
(d) use of interpretive forums (described above).

The study recognises the need for co-development of outputs so that different interests, values and subject positions can be accommodated.

Standards for Reporting Qualitative Research (SRQR)[xxxv] have been followed in the protocol design.

ACKNOWLEDGEMENTS

FUNDING

This work is supported by the National Institute of Health Research [NIHR], Health Service and Delivery Stream, grant number HS&DR 17/99/85. The study has been adopted by the NIHR Applied Research Collaboration, South London.

REGULATORY APPROVALS

Phase 1 study approval was granted by King's College London University Ethics Panel [BDM RESC 11174]. Approvals for Phase 2 and 3 was gained through the Integrated Research Application System application and by Research Ethics Committee and by Confidentiality Advisory Group precedent set category 10 application) scrutiny [IRAS:262197]. Overall study sponsorship is by King's College London (HS&DR 17/99/85).

AUTHOR CONTRIBUTIONS

MA drafted the protocol with co-ordinated ongoing contributions from JS, RI, AH, MT, MB and CB. JH assisted with the proof reading the protocol for submission for publication.

For peer review only - http://bmjopen.bmj.com/site/about/guidelines.xhtml

COMPETING INTERESTS
The authors declare that they have no competing interests. The views and opinions expressed therein are those of the authors and do not necessarily reflect those of the HS&DR program, NIHR, NHS or Department of Health.

---

[i] Berlinger, N. and Wu, A.W., 2005. Subtracting insult from injury: addressing cultural expectations in the disclosure of medical error. *Journal of Medical Ethics*, *31*(2), pp.106-108.
[ii] Berlinger, N. 2005. *After Harm: medical error and the ethics of forgiveness* (Vol. 18). Baltimore, MD: Johns Hopkins University Press
[iii] Wu, A.W., McCay, L., Levinson, W., Iedema, R., Wallace, G., Boyle, D.J., McDonald, T.B., Bismark, M.M., Kraman, S.S., Forbes, E. and Conway, J.B. 2017. Disclosing adverse events to patients: international norms and trends. *Journal of Patient Safety*, *13*(1), pp.43-49.
[iv] Vincent, C. and Amalberti, R. 2016. *Safer Healthcare.* Cham: Springer International Publishing
[v] Piper D, Iedema R. *Literature Review: Incident Disclosure Policy, Legal Reform and Research Since 2008.* Sydney: Centre for Health Communication (University of Technology) and Australian Commission on Safety and Quality in Health Care; 2011
[vi] Iedema, R., Allen, S., Britton, K., Piper, D., Baker, A., Grbich, C., Allan, A., Jones, L., Tuckett, A., Williams, A. and Manias, E. 2011. Patients' and family members' views on how clinicians enact and how they should enact incident disclosure: the "100 patient stories" qualitative study. *BMJ*, *343*, p.d4423.
[vii] Iedema et al 2011 (above)
[viii] https://www.professionalstandards.org.uk/what-we-do/improving-regulation/find-research/duty-of-candour
[ix] Action Against Medical Accidents. 2016. Regulating the duty of candour: *A Report by Action Against Medical Accidents on CQC Inspection Reports and Regulation of the Duty of Candour*. August 2016
[x] Birks Y, Harrison R, Bosanquet K, R. Iedema. 2014. An exploration of the implementation of open disclosure of adverse events in the UK: a scoping review and qualitative exploration. *Health Services and Delivery Research. 2.20.*
[xi] https://www.professionalstandards.org.uk/what-we-do/improving-regulation/find-research/duty-of-candour
[xii] National Patient Safety Agency *Being Open: Communicating Patient Safety Incidents*. National Reporting and Learning Service. November 2009.
[xiii] *Care Quality Commission.* 2017a. *The Statutory Duty of Candour* www.cqc.org.uk/guidance-providers/regulations.../regulation-20-duty-candour downloaded 15.02.18.
[xiv]Cumberlege, J. 2016. National Maternity Review. Better Births. London: NHS.
[xv] Department of Health. The National Maternity Safety Strategy: Progress and Next Steps. November 2017
[xvi] NHS Resolution: The Early Notification Scheme Progress Report: collaboration and improved experience for patients. https://resolution.nhs.uk/resources/early-notification-scheme-progress-report/
[xvii] Wong, G., Westhorp, G., Pawson, R. and Greenhalgh, T. 2013. Realist synthesis: RAMESES training materials. *London: University of London*.
[xviii] Pawson, R. 2006. *Evidence-based policy: a realist perspective*. Sage.
[xix] Pawson, R., Greenhalgh, T., Harvey, G. and Walshe, K. 2004. Realist synthesis: an introduction. *Manchester: ESRC Research Methods Programme, University of Manchester*.
[xx] Pawson, R. and Manzano-Santaella, A. 2012. A realist diagnostic workshop. *Evaluation*, *18*(2), pp.176-191.
[xxi] Manzano, A. 2016. The craft of interviewing in realist evaluation. *Evaluation*, *22*(3), pp.342-360.
[xxii] Mendizabal E. 2012. *The Alignment, Interest and Influence Matrix (AIIM).* Overseas Development Institute and Research and Policy in Development
[xxiii] Leslie, M., Paradis, E., Gropper, M.A., Reeves, S. and Kitto, S. 2014. Applying ethnography to the study of context in healthcare quality and safety. *BMJ Qual Saf*, *23*(2), pp.99-105.
[xxiv] Dixon-Woods, M., Leslie, M., Tarrant, C. and Bion, J. 2013b. Explaining Matching Michigan: an ethnographic study of a patient safety program. *Implementation science*, *8*(1), p.70
[xxv] Lawton, R., Taylor, N., Clay-Williams, R. and Braithwaite, J., 2014. Positive deviance: a different approach to achieving patient safety. *BMJ quality & safety*, *23*(11), pp.880-883.
[xxvi] Stake, R. E. 2008. Qualitative case studies. In N. K. Denzin & Y. S. Lincoln (Eds.), *Strategies of qualitative Inquiry* (p. 119–149). Sage Publications, Inc.
[xxvii] Argyris C. 1993. *Knowledge in Action: A Guide to Overcoming Barriers to Organisational Change*. California Josse-Bass.
[xxviii] Spencer L., Ritchie J., O'Conner 2003 Analysis: Practices, Principles and Processes in Ritchie J. and Lewis J. *Qualitative Research Practice*. London: Sage.
[xxix] Rycroft-Malone, J., Burton, C.R., Wilkinson, J., Harvey, G., McCormack, B., Baker, R., Dopson, S., Graham, I.D., Staniszewska, S., Thompson, C. and Ariss, S. 2015. Collective action for implementation: a realist evaluation of organisational collaboration in healthcare. *Implementation Science*, *11*(1).
[xxx] Mendizabal E. 2012. Above.
[xxxi] Hinton, T., Gannaway, D., Berry, B. and Moore, K., 2011. The D-cubed guide: Planning for effective dissemination.
[xxxii] Rakime E. 2011. Interviewing people about potentially sensitive topics. Nurse Researcher (through 2013), 19(1).
[xxxiii] Razzaghi, A. and L. Afshar 2018. Ethical issues related to data collection methods in trauma researches. Medical Ethics Journal, 11 (42), pp.7-10.
[xxxiv] https://www.invo.org.uk/posttypepublication/national-standards-for-public-involvement/
[xxxv] O'Brien BC, Harris IB, Beckman TJ, Reed DA, Cook DA. **Standards for reporting qualitative research: a synthesis of recommendations.** *Academic Medicine*, Vol. 89, No. 9 / Sept 2014 DOI: 10.1097/ACM.0000000000000388

For peer review only - http://bmjopen.bmj.com/site/about/guidelines.xhtml

Study Flow Diagram: Strengthening Disclosure in Maternity

- PURPOSIVE SAMPLING AND APPROACH FOR CASE STUDY SITES

HS&DR Study Number:17/99/85. **IRAS ID: 262197**

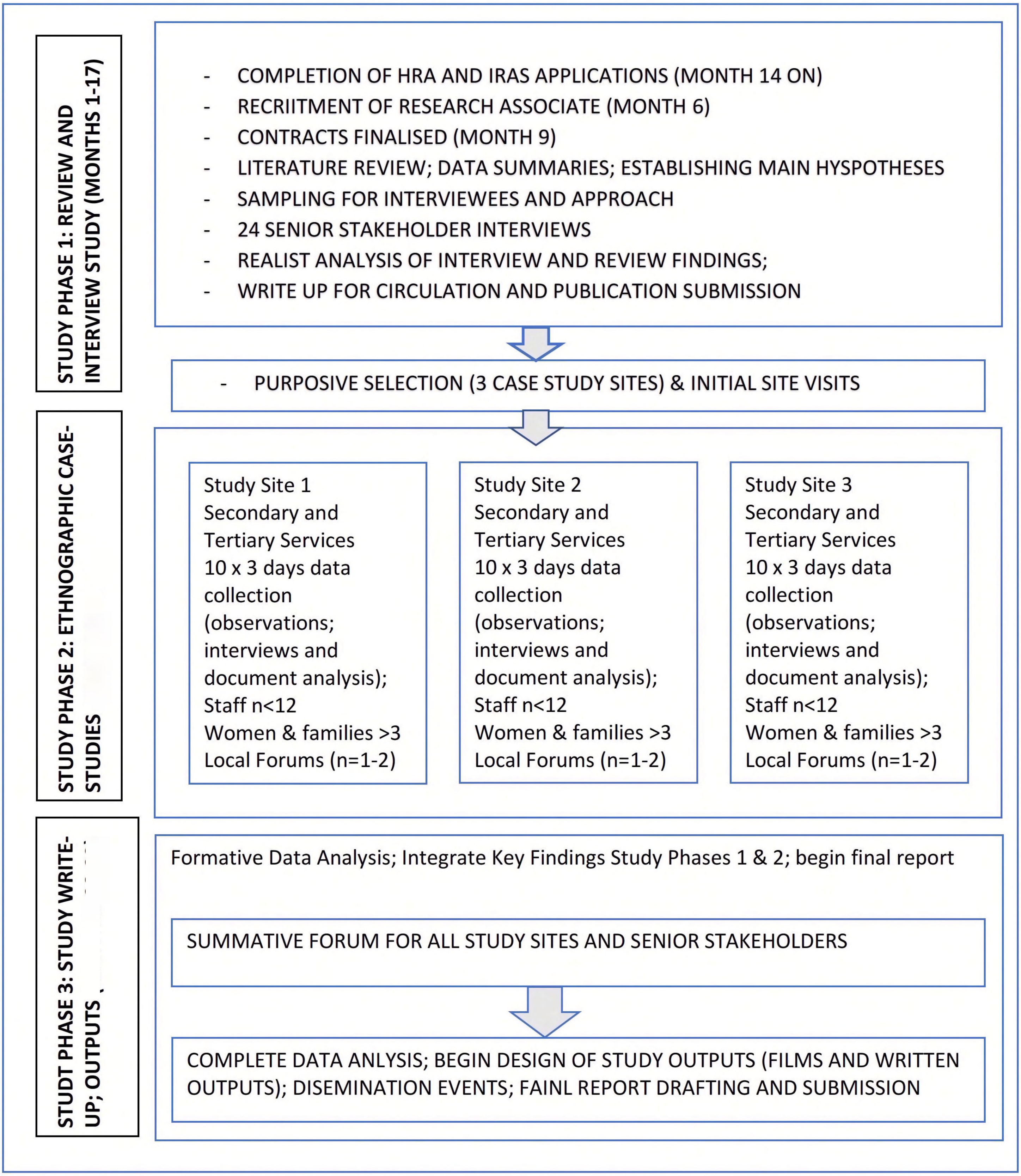

Appendix 2:

Study Recruitment And Data Management For Study Phase 1b

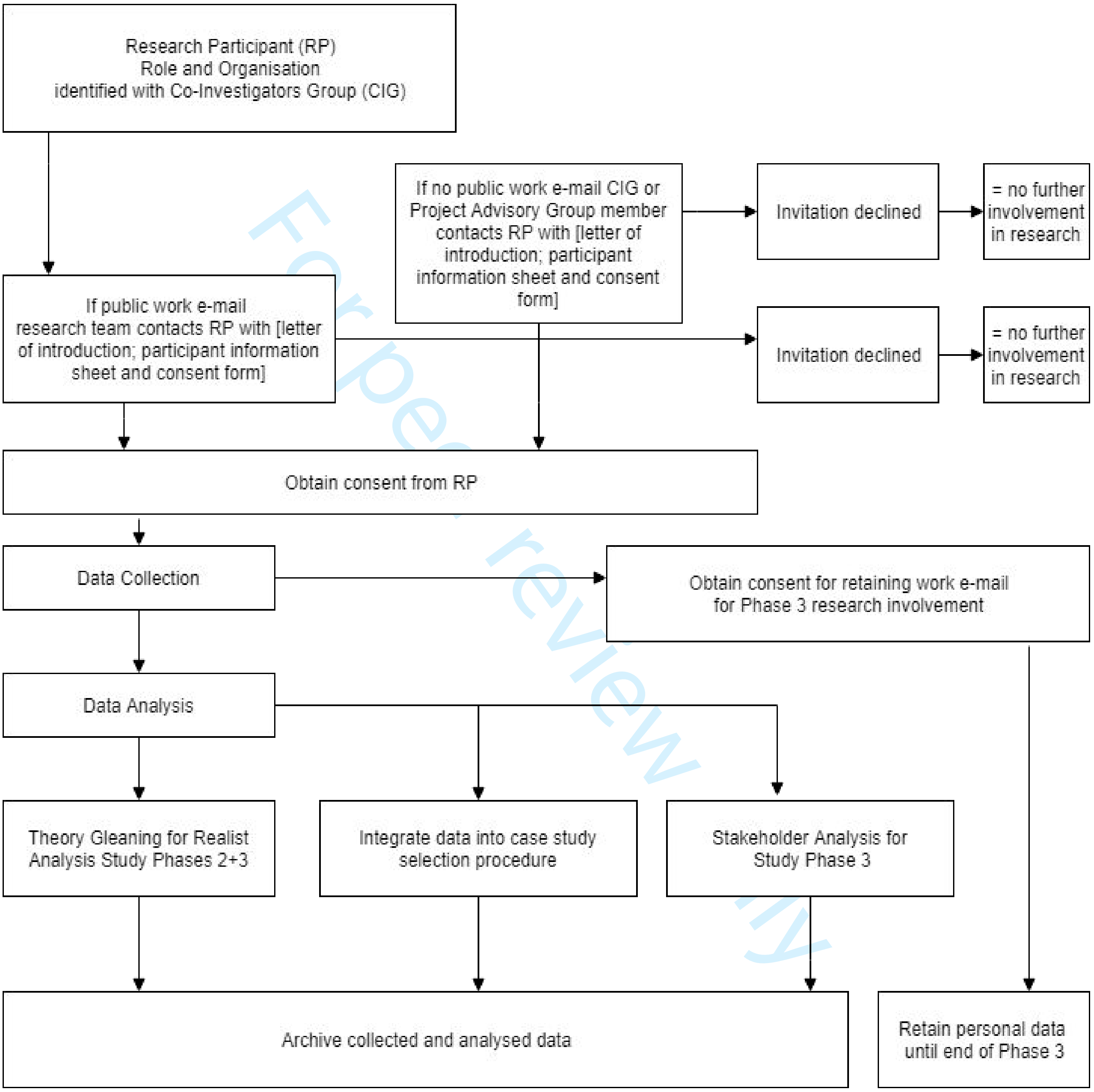

APPENDIX 3: STRATEGY FOR SHORT-LISTING POTENTIAL CASE STUDY SITES

4 potential study sites have been identified using a staged sampling frame to identify a purposive sample of organisations or services that are undertaking improvement work in OD in NHS maternity care. We have considered maternity networks, trusts or service areas or services as potential study sites. The staged sampling frame has been two-staged:

Stage 1:

Long listing potential study sites from public data sets (notably trust-level scores on 'Transparency: Learning from Mistakes League' and the NHS staff survey). This data is reported annually and shows variations between all NHS acute trusts (from outstanding levels of openness and transparency [level 1] to poor reporting culture [level 4]. This data set is established from selected metrics from the annual NHS staff survey and from the National Reporting and Learning System. Service-level scores from the annual NHS staff survey, '*Reporting on 'Errors and Incidents', selected items* [KF 29; 30;31] (see Box 1).

Box 1: 'Errors and Incidents': items from the annual NHS staff survey (by trust and location)

**Survey Item**	**Error and Incidents: by Trust and by Trust Location (Women's Health)**
KF 29	staff reported errors, near misses or incidents witnessed in last month (percentage score)
KF 30	staff reported fairness and effectiveness of procedures for reporting errors, near misses and incidents (scale summary score)
KF 31	staff reported confidence and security in reporting unsafe clinical practice (scale summary score)

These key findings are benchmarked against others trusts and for different locations [or divisions] within that trust. Women's Health, Gynaecology and Obstetrics, as well as Midwifery are all locations. Therefore, we have been able to establish a comparative picture of how staff experience incident investigation, across and between our listed sites in these areas and listed acute trusts.

We have long-listed those trust and service areas where there is good or improving performance reported in fields that indicate candour (for example, transparency, incident reporting, staff reported organisational or service responsiveness of incident reporting).

Stage 2:

Short-listing long-listed sites using combined soft intelligence from national and regional stakeholders (at Phase 1 scoping study).

Due to the sensitivity of the research topic for frontline staff as well as for services and trusts, and the anticipated challenges of organisational engagement in some trusts, services and networks, our sampling frame follows Stake's (2013) principles to:

i. maximise chances of ongoing engagement of participants in the research
ii. provide diversity of cases across contexts
iii. ensure that all cases provide good opportunities to learn about complexity and context

For peer review only

Therefore, we shortlisted only those services, trusts and networks identified as potentially available as case study sites by stakeholder or PAG recommendation.

Reference:

Stake R. E. 2013. *Multiple Case Study Analysis*. Guildford Press.

For peer review only

Appendix 4: Recruitment strategy for organisations and Individuals.

Sample Identification for Study Phase 2

Study Phase 2 (up to 4 case studies, with each case study comprising focused (group or individual staff) non-participant observations (n=25 hours); in-depth interviews with NHS staff and patients (approximately n=12); and collection and analysis of redacted documentary data (n=20 hours). Some return interviews may be arranged with staff who lead the OD improvement work (n=approximately 3 per site).

Recruitment of case study sites into the study will involve a range of approaches in order to maximise opportunities of team, service, trust and/or network engagement. These are:

a. sampling strategy for the potential case study sites:
The identification of any site for potential recruitment will be on the basis of their already being active in maternity quality and safety improvement work and on the basis of their likely interest in engaging with the study team (as identified by Phase 1 interviewees and our independent project advisors). We will therefore ensure that prior to our sampling for diversity or context, we have sampled to maximise our chances of ongoing engagement of services, trusts and/or networks in the study. In addition, we will identify potential sites by internet searches. These searches will assist in our closer identification of maternity safety and learning 'candour' collaborative or working groups (for example, as developed in the Health Innovation Networks or as supported by local NHS staff or parent/maternity support groups).

b. use of a recommended framework for enhancing study participant recruitment:
We understand that the recruitment of organisations and networks, as well as of those who work within them, is a multi-layered process. We will draw on McDonald's (2011) adapted business-model approach to recruitment to inform our recruitment plan.

We will undertake:

i. **Early and widespread familiarisation of the research project (the study question, along with the ongoing promotion of study value, legitimacy and potential benefits) across NHS maternity services.** We will initiate promotion of the study (and of the senior researchers involved) from study start date. This promotion will be established and sustained through social media (Twitter; a project blog and, on the advice of our independent project advisors, a study update newsletter) until project completion. Prior to Phase 1, and after the study has been contracted, our study team and independent project advisors will be encouraged to 'spread the word' on the research through regional and national networks. This familiarisation and ongoing promotion will be underpinned by a simple message of 'what the study stands for' and how it can be recognised.

ii. **Early identification of potential case study sites.** We will be anticipating potential sites throughout the Phase 1 research. This will include establishing short 'case study' profiles to aid us with sampling following Stake's (2013) case study sampling principles as well as by making early contact with potential sites through gatekeepers identifiable on public websites. These initial contracts (and a response after one email contact and one email reminder) will aid our assessment of their interest in potential participation.

iii. **Anticipation of multiple dimensions of recruitment**. We are aware that access to services, trusts and/or networks will require successful engagement with multiple sponsors and gatekeepers, both formally and informally. We will use tailored

messages to engage different interest groups (for example, the potential benefits of involvement in the study for patient representatives will differ from those for a group of junior clinicians or managers). We will also identify the range of influential sponsors, champions and change agents within potential case study sites to enlist their ongoing support.

iv. **Assurance to potential participants (organisations and individuals) of the scope and limits of the research across and within case study sites.** Macdonald (2011) highlights the necessity of clarifying the expectations of organisational participation and of the scope and limits of the research to be undertaken to all gatekeepers. This will be particularly important for the case-studies as participants or gatekeepers will be concerned that they may have less control over information shared through survey or interview methods. We will reiterate in all communication materials, that our non-participant observations are focused on the contexts and routines that make OD possible and that we will not be expecting to observe actual OD meetings with women or families (see case-study methods described below). We will not be recruiting women or families from the case study sites as study participants.

v. **Emphasis on the actual and potential benefits of research participation to key gatekeepers as well as to individual study participants**. We are assuming that there will be some reluctance of some potential participants to engage with this study topic. However, we have sought to enhance to benefits to participants by our study design (Phase 3 offers opportunities for networking, social interaction and sharing experiences both within the sites, from our Women and Family Forums and from our Summative Interpretive Forum). Our communications will highlight these opportunities.

vi. **The engagement of staff as potential and actual research participants will be facilitated by short presentations of the study and key study messages by the researchers at local unit meetings in different service areas**. By identified quality and safety improvement champions and by snowballing through clinical or project teams. The ongoing development of ongoing research relationships, with two named and experienced health service ethnographers, will further support recruitment processes.

For peer review only

INVESTIGATION OF THE CRITICAL FACTORS REQUIRED TO IMPROVE THE DISCLOSURE AND DISCUSSION OF HARM WITH AFFECTED WOMEN AND FAMILIES: STUDY PROTOCOL FOR A QUALITATIVE, REALIST STUDY IN NHS MATERNITY SERVICES [THE DISCERN STUDY].

Journal:	*BMJ Open*
Manuscript ID	bmjopen-2020-048285.R1
Article Type:	Protocol
Date Submitted by the Author:	09-Dec-2021
Complete List of Authors:	Adams, Mary; King's College London, Department of Women and Children's Health, Faculty of Life Science and Medicine, King's College, London. 10th floor. North Wing, St. Thomas' Hospital. Westminster Bridge Road, London SE1 7HE<br>Iedema, Rick; King's College London, Centre for Team-Based Practice and Learning in Health Care, Guy's Campus. London SE1 1UL<br>Heazell, Alexander; University of Manchester, Maternal and Fetal Health Research Centre<br>Treadwell, Maureen; Birth Trauma Association<br>Booker, Maria; Birthrights<br>Bevan, Charlotte; Sands, stillbirth and neonatal death society<br>Hartley, Julie; King's College London, Department of Women's and Children's Health, Faculty of Life Science and Medicine, King's College, London. 10th floor. North Wing, St. Thomas' Hospital. Westminster Bridge Road, London SE1 7HE<br>Sandall, Jane; King's College London, Department of Women and Children's Health, Faculty of Life Science and Medicine, King's College, London. 10th floor. North Wing, St. Thomas' Hospital. Westminster Bridge Road, London SE1 7HE
**Primary Subject Heading**:	Qualitative research
Secondary Subject Heading:	Communication, Health services research, Research methods, Obstetrics and gynaecology
Keywords:	Quality in health care < HEALTH SERVICES ADMINISTRATION & MANAGEMENT, Organisation of health services < HEALTH SERVICES ADMINISTRATION & MANAGEMENT, Maternal medicine < OBSTETRICS, Protocols & guidelines < HEALTH SERVICES ADMINISTRATION & MANAGEMENT, Health policy < HEALTH SERVICES ADMINISTRATION & MANAGEMENT

SCHOLARONE™
Manuscripts

INVESTIGATION OF THE CRITICAL FACTORS REQUIRED TO IMPROVE THE DISCLOSURE AND DICUSSION OF HARM WITH AFFECTED WOMEN AND FAMILIES: STUDY PROTOCOL FOR A QUALITATIVE, REALIST STUDY IN NHS MATERNITY SERVICES [THE DISCERN STUDY].

Dr Mary Adams, Professor Rick Iedema, Dr Alexander Heazell, Ms Maureen Treadwell, Ms Maria Booker, Ms Charlotte Bevan, Dr Julie Hartley and Professor Jane Sandall

Corresponding Author:
Dr Mary Adams
Department of Women and Children's Health
Faculty of Life Science and Medicine, King's College London
10th floor. North Wing.
St Thomas' Hospital
Westminster Bridge Road
London SE1 7HE.

mary.adams@kcl.ac.uk
Tele: 07717473227

Co-Author Details:
Professor Rick Iedema, King's College, London. England.
Professor Alexander Heazell, University of Manchester. England.
Ms. Maureen Treadwell, Birth Trauma Association. England.
Ms. Maria Booker, Birthrights. England.
Ms. Charlotte Bevan, Stillbirth and Neonatal Death Charity. England
Dr. Julie Hartley, King's College, London. England.
Professor Jane Sandall, King's College, London. England.

Key Words:
TRUTH DISCLOSURE
PROFESSIONAL-PATIENT RELATIONS
PATIENT SAFETY
ORGANISATION OF HEALTH SERVICES
PROGRAM EVALUATION

Word Count (excluding title page, references, figures and tables): 3,354

For peer review only

INVESTIGATION OF THE CRITICAL FACTORS REQUIRED TO IMPROVE THE DISCLOSURE AND DISCUSSION OF HARM WITH AFFECTED WOMEN AND FAMILIES: STUDY PROTOCOL FOR A QUALITATIVE, REALIST STUDY IN NHS MATERNITY SERVICES [THE DISCERN STUDY].

ABSTRACT

Patients and families are entitled to disclosure and discussion of healthcare incidents affecting them. This reduces distress and contributes to learning for safety improvement. Complex barriers prevent effective disclosure and continue in the English NHS, despite a legal duty of candour. NHS maternity services are the focus of significant efforts to improve this. There is limited understanding of how, and to what effect, they are achieving this.

METHODS and ANALYSIS

A 27-month, three-phased realist evaluation identifying the critical factors contributing to improvements in the disclosure and discussion of incidents with affected families. The evaluation asks 'what works, for whom, in what circumstances, in why respects and why?'.

Phase 1: establish working hypotheses of key factors and outcomes of interventions improving disclosure and discussion, by realist literature review and in-depth realist interviews with key stakeholders [n=approximately 20]

Phase 2: refine or overturn hypotheses, by ethnographic case-study analysis using triangulated qualitative methods (non-participant observation; interviews [n=12]; documentary analysis) in up to 4 purposively-sampled NHS trusts.

Phase 3: consider hypotheses and design outputs during 7 interpretive forums.

ETHICS AND DISSEMINATION:

Phase 1 study approval by King's College London Ethics Panel [BDMRESC 22033]. National Research Ethical Approval for Phases 2-3: [IRASID:262197](CAG:20/CAG/0121)(REC:20/LO/1152). Study sponsorship by King's College London (HS&DR 17/99/85).

Findings to be disseminated through tailored management briefings; clinician and family guidance (written and video); lay summaries, academic papers, and report with outputs tailored to maximise academic and societal impact. Views of women/family groups are represented throughout.

STUDY STRENGTHS AND LIMITATIONS:

- The first investigation of interventions supporting disclosure in NHS maternity care.
- Methodology includes multi-disciplinary co-design throughout the research cycle, with wider applicability for health service research.
- A realist approach examines various contexts, mechanisms and outcomes of intervention success.
- Research does not examine disengagement among some maternity providers.

For peer review only - http://bmjopen.bmj.com/site/about/guidelines.xhtml

INTRODUCTION

Besides patients' entitlement to the disclosure of clinical incidents affecting them[1] when disclosure processes are conducted well, they can reduce the emotional harm to all involved[2], provide valuable opportunities for learning for improvement[3, 4] and may reduce public or private expenditures on investigatory processes and legal and financial claims[5]. In the longer-term, disclosure practices may revise patient-clinician hierarchies and/or encourage public trust in healthcare[6].

However, there is often disagreement or uncertainty over the principles, purpose and impact of disclosure practices. Their enactment often falls short of the expectations of harmed individuals[6], professional or organisational guidelines[7], and regulatory directives[8]. In the UK, fears of professional investigation, loss of reputation, and blaming behaviours are common deterrents to more open communication with patients. Inflexible organisational procedures and limited investment in the training and support of clinicians undertaking disclosure and discussions with patients can mean that the needs of patients are not properly heard or responded to[9].

In the English NHS, each clinician's professional duty of honesty[7] co-exists with 'Being Open' policy guidance[10] that is now underpinned by a statutory duty of candour on health providers[11]. In NHS maternity services, an array of national and local safety improvement programmes is ongoing to reduce safety incidents and to improve the care of women/families when serious incidents happen. Since 2017, in England, there have been further efforts to improve incident investigation and women/family involvement in these processes[12, 13]. One driver for these interventions is the reduction of the escalating costs of clinical negligence claims in NHS maternity care[14]. There is limited understanding of how, in what situations, to what effect and for whom these various improvement programmes are being implemented in maternity services and service areas, and their consequence for women/families, clinicians and organisations. Evidence is required to understand which improvements address these different groups' needs and interests when a harmful incident happens and how.

The primary aim is to develop actionable evidence to inform maternity providers of those factors that, in particular situations, will improve the disclosure and discussion of adverse incidents in NHS maternity care with affected women/families. The secondary aim is the co-production of outputs (support materials for women/families, clinicians and managers, and practical recommendations for organisations) to benefit these groups during these events. The overarching research question is 'what are the critical factors that can improve the incidence and quality of Open Disclosure in NHS maternity services?'. We will explore the actual or anticipated consequences of ongoing improvement programmes for different participants in particular contexts.

The study is co-designed and co-produced with three national Patient and Public Involvement and Engagement (PPIE) partners, including charities and associations representing women/families who have experienced harm in maternity care. These partners are core members of our Co-Investigator Group (CIG). The work is also informed by a wider Project Advisory Group [PAG] that includes eight individual women and families with direct experience of harm in maternity care. The PAG advises on study progress, outputs and dissemination.

METHODS AND ANALYSIS

Study Design

The ongoing interventions in NHS maternity care comprise policy-led, complex interventions promoted in services in the expectation that incidence and/or quality of women/family involvement in the disclosure and discussion of adverse events will be improved. A realist, theory-driven evaluation approach informs study design. In health service evaluations, realism examines the interaction between a complex social intervention or 'programme', the contexts of strategic and

For peer review only - http://bmjopen.bmj.com/site/about/guidelines.xhtml

everyday implementation work, and the un/intended outcomes for different participants[15]. Realist evaluation asks, 'what works, for whom, in what circumstances, in what respects and why? '[16]. Therefore, the approach does not seek to establish the overall effect of an intervention but to identify the critical factors (in realist terms, the 'generative mechanisms') that are likely to produce expected and unexpected effects for different groups in particular circumstances[17]. The approach enables the analysis of the non-linear, contingent qualities of interventions, including the situated conditions of success and failure.

Realist evaluation follows a staged approach that moves from identifying initial working and interconnected hypotheses from secondary sources to the 'testing' (refinement or overturning) of these hypotheses using primary data to identify those contingencies that drive and direct improvement[18].

The 3-phased, 27-month, qualitative study will:

- establish a series of working hypotheses by literature scoping and expert stakeholder consultation (Study Phase 1a).
- 'test' these working hypotheses by in-depth interviews with a range of national stakeholders (Study Phase 1b) and by ethnographic research (Study Phase 2).
- agree with national stakeholders and different interest groups and experts, the critical factors required to trigger and sustain interventions to improve the disclosure and discussion of harm with women/families in NHS maternity care; co-design outputs to support these generative factors (Study Phase 3).
- The study will follow the quality standards for realist evaluation[19].

The study runs from May 2019 to April 2022 with 9 months COVID-related research interruption.

See Appendix 1 for the DISCERN study flow diagram.

Methods for each study phase

Phase 1a

The PROSPERO registered realist review will identify and extract literature from electronic databases from social science, nursing, and medical and health service management. The purpose of the review is descriptive and will identify papers and documents of relevance to disclosure and discussion of harm in international maternity services. Initial scoping searches will identify keywords, synonyms, spelling variations. Searching will include free text and database-specific subject headings (such as MeSH terms), advanced Boolean truncation, 'explode' and other techniques. The database search will be supplemented by 'snowball' strategies, scanning reference lists from relevant papers and advice from subject experts. Inclusion criteria will be 'maternity and perinatal services' and 'disclosure and discussion of harm'. Following realist methodology for data gathering[20], no pre-determined exclusion criteria concerning research methods will be applied. Grey literature, including policy reports, service guidance and public and professional commentary, will be retrieved. The search is limited to English language documents. Identified full-text papers will be rescreened for both inclusion and exclusion criteria and relevance and rigour. If over 50 papers are identified, those most relevant to the emerging programme theories will be selected. A structured realist data extraction form will be developed to document item; context; intervention details/indications of mechanism/outcome relationships; factors affecting implementation; evidence of improvement program outcomes.

Following RAMESES guidance[19], a realist analysis of extracted data will develop Context-Mechanism-Outcome configurations. From these, and with our stakeholders, we will identify initial (Phase 1a) programme theories and potential middle-range theories for examining the critical factors that

For peer review only - http://bmjopen.bmj.com/site/about/guidelines.xhtml

influence improvements and their consequences for women/families, clinicians and services. One-to-one discussion in the CIG and consultation with the PAG, comprising academics, policymakers, patient and public advocates and front-line clinicians, will develop and validate our secondary data analysis.

Phase 1b

One-to-one, open-ended, audio-recorded telephone interviews with approximately 20 national stakeholders in service improvement and patient/family engagement in NHS maternity care using a topic guide developed from Phase 1a findings and piloted with co-investigators. This interview study will (i) explore interviewees' various experiences and views concerning the programme theory developed from the realist review[21]; (2) gather 'soft intelligence 'on site selection for Study Phase 2; (3) establish stakeholders' views on how to maximise research impact[22] for Study Phase 3.

Interviewees will be sampled for maximum diversity across national stakeholder groups. Advice from the CIG and the PAG and snowball sampling with interviewees will guide the interviewee approach and selection.

See Appendix 2 for the flow diagram of study recruitment and data management for Study Phase 1b.

Phase 2

Up to 4 NHS maternity services, identified as high-performing or improving (in terms of supporting interventions for strengthening disclosure practices with women/families after harm in maternity care), will be selected as ethnographic case studies. An ethnographic methodology will enable in-depth interpretation of the context and practices of clinical or organisational relationships, including the situated and often taken-for-granted qualities of these practices, including their negotiation and legitimation[23]. This approach is important for understanding the local reception, adjustments and viability of new interventions[24]. Ethnographic case studies enable detailed data to be analysed dynamically regarding particular organisational and service contexts, events and activities, and as a series of comparative case studies.

A case study comprises 10 x 3-day visits, completed over ten months, with periods between visits for researchers to discuss and compare emergent findings. Initial hypotheses and their refinements or revisions (developed in Study Phase 1) will focus the comparative case studies. However, they will also document (i) how relevant policy and procedures are promoted, managed and monitored as well as interpreted and evaluated by recipients (along with the challenges and opportunities to this);(ii) factors that influence the 'when and how' of procedural enactments in actual clinical settings by different participants including women/families, clinicians, administrators and management and governance teams; (iii) the interplay of formal and informal local workplace cultures and wider public and policy debates; (iv) personal, team and service histories if disclosure experiences, including their potential to influence wider revisions in the 'Being Open' agenda, patient-clinician-manager relationships and their acceptance as routine.

Our sampling follows social science principles. Due to expected challenges in engaging managers or clinicians in some maternity services, the sampling frame includes only cases of positive deviance to maximise engagement in the study. A positive deviance approach holds that behaviours (and situations) the produce poor practice or experience are variations on the same processes that produce successful practices. Therefore, focusing on success is a more effective tactic for understanding how improvements are achieved[25]. Sampling includes maximum diversity between services so that opportunities to learn about in-case and cross-case variation is maximised.[26] A short-listing of potential case study sites will use public data set scores; information on service provision (size, population served, neonatal care provision).

For peer review only - http://bmjopen.bmj.com/site/about/guidelines.xhtml

Further information on the sampling framework is described in Appendix 3.

Approach and recruitment of selected organisations, site Principal Investigators [PIs], and key gatekeepers, is tailored from a theory-based, business-model approach to recruitment to clinical studies. A description of the application of this business model to the study is described in Appendix 4.

Each ethnographic case study will triangulate findings collected by the following methods:

> *Focused Observations* [estimated 25 hours] of formal and informal events and routines intended to secure improvement in disclosure of adverse incidents with women/families (e.g. service-level and Trust-level meetings on serious incident reporting and analysis; training activities; promotion and publicity work; and meetings between patient liaison and clinical teams. Observations may include meetings between family members and organisational representatives.
>
> *In-depth Return Interviews* with about 12 staff [some at two time-points] involved with, or affected by, the improvement work (e.g. clinical and governance leads; patient support personnel; more junior clinicians).
>
> *Documentary Data* including redacted incident reports; meeting minutes, action plans; organisational and service bulletins; training reports; quality and safety improvement reports; and incident reports from families to construct a retrospective picture of OD work within that case study.

With participants' informed consent, in-depth interviews will be audio-recorded on encrypted devices and transcribed verbatim. The observational data will be recorded as low inference notes and later word-processed by that researcher. All documentary data will be redacted before inclusion in the study.

Phase 3

Following initial analysis of case-study data, seven forums of purposively-selected individuals will validate findings[27] and co-design, with the CIG, training materials from the study findings. Audio-recorded forum discussions will be thematically analysed to (a) further validate and develop findings on the critical factors and situations that support disclosure processes; (b) support output co-development.

Potential participants will be identified during participation with Phase 1 or 2 and sampled for maximum variation in their experiences of disclosure practice improvements. A maximum of 25 participants will be invited to any one of the seven local forums. A final national forum, including local forum representatives and national policymakers, will accommodate up to 60 participants and be externally facilitated. The national event will be hosted at the sponsoring university. Wider consultations of study findings and output development will be with established online forums for harmed patients/families and clinicians interested in maternity safety improvement.

Public and Patient Participation and Engagement

To ensure that the views and interests of women/families, and the wider public, remain the focus of the study, our PPI strategy supports representation at all study phases (study design, management, data interpretation, co-development of outputs, reporting of findings and dissemination of research

For peer review only - http://bmjopen.bmj.com/site/about/guidelines.xhtml

findings, along with other outputs). We have followed the new national standards[32] which provide a series of indicators to support structures and behaviours that support PPI in study governance and study impact. The approach reflects current policy emphasis on participatory approaches to healthcare service quality and safety improvement. This is to be achieved by:

- The involvement of women/families and their charity and association representatives in all stages of the research cycle (study application, research planning, research activity, study steering, write-up and dissemination of findings).
- A multi-disciplinary and multi-sector research team that includes public members.
- Ongoing collaboration with an extensive and varied PAG, including their involvement in the summative forum.
- Use of interpretive forums (described above).

The study recognises the need for co-development of outputs so that different interests, values and subject positions can be accommodated. Standards for Reporting Qualitative Research (SRQR)[33] have been followed in the protocol design.

DATA ANALYSIS

Data will be organised and analysed following a realist logic of analysis. Retroductive analysis (moving between inductive and deductive approaches) will identify and explore main Context-Mechanism-Outcome patterns, particularly the generative causal mechanisms shaping these configurations. This approach is to refine, develop or refute initial program theories developed progressively from Study Phase 1 (realist literature review) and Study Phase 2 (ethnographic case studies). Additional C-M-O configurations may be elucidated at Study Phase 2 and examined retroductively across this data set to inform, revise or add the program theories. Data analysis will be conducted at the close of each study phase to maintain an ongoing and iterative approach to programme theory refinement. Two researchers trained in realist evaluation will independently identify and explore main C-M-O configurations. Then with additional researchers and stakeholders, explore programme theory refinement in the light of these. Phase 2 interview, ethnographic and documentary data will also be analysed in a triangulating fashion to develop concrete descriptions of the *generative mechanisms* underlying OD interventions and the contextual factors that trigger effects at particular times. We will seek to identify convergent and contradictory evidence within and then across the interventions' different organisational, interpersonal and individual contexts. Coding and data management will be organised and audited using qualitative data analysis software, Nvivo (V12), and CMO data coding sheets will be developed for the analysis.

Where possible, cross-tabulations and scattergrams will be used to plot the relationship between apparently anomalous aspects of improvement work in this area and other social variables (e.g. distinctive service or organisational histories, resourcing agreements, or public involvement structures and relationships).

A stakeholder analysis using the RAPID AIIM matrix[22] may be used to assist in mapping participant interests in the subject area and their influence over local and national improvement work.
The hypotheses generated will also be developed using conceptual frameworks drawn from the social sciences (for example, Normalisation Process Theory, an Institutional Logics approach, approaches to trust in health care organisations; and perspectives on team and organisational safety and resilience. RAMESES II reporting standards for realist evaluations will be followed for the presentation of data collection, findings and discussion[28]

ETHICS AND DISSEMINATION

For peer review only - http://bmjopen.bmj.com/site/about/guidelines.xhtml

Outputs will be robust and actionable evidence about the organisational strategies. These team, service and network practices can support and enhance the quality and extent of OD within NHS maternity care and wider NHS care provision within particular circumstances and with respect to different interests groups. Our findings are intended to impact three organisational areas of health care: the micro-level of disclosure processes and relationships; meso-level of service management and organisation of these processes; and macro-level of policy. Therefore, our dissemination strategy, with outputs tailored to the audience[29], includes:

- Co-designed briefing documents and guidance for maternity service managers and policymakers.
- Co-designed information materials (likely, short guidance sheets and video animations for services, clinicians and women/families (and promotion of these by our Stage 3 study participants).
- Accessible public information and lay summaries of study findings disseminated through our dedicated Twitter account and website.
- Peer-reviewed academic journal articles (including the final NIHR study report)

A summary of findings sent to all study participants who have consented to receive this information. More widely, the dissemination plan is underpinned by all RCUK identified factors that help to generate academic and societal impact. The research will enhance the evidence base for improving the use and quality of OD in NHS maternity care, influencing policy and practice; produce theoretically informed knowledge of the critical factors for fostering, enhancing and sustaining OD; and will support the growth of networks and knowledge exchange between NHS maternity providers, women/family interest groups and associations, OD policy-makers and non-maternity NHS providers.

Ethical Considerations

We have identified this study as a high risk, qualitative research study because we are:

- Conducting research on a highly sensitive and potentially emotive topic for all concerned.
- Interviewing and observing clinicians and managers whose care might have resulted in harm.
- Observing meetings with women/families and managers that may become adversarial.

The primary ethical issues requiring mitigation are:

- *Non-consent or withdrawal of consent to study participation* (for example, by some staff during research observation of staff meetings and routines). In these situations, we will always respect the withdrawal of study participation and state this in all participant information and consent forms. We also will seek to focus observations of collective routines on more public workplace activities (e.g. training events)
- *Disclosure of negligence* (for example, during staff and women/family representative interviews). We will make it clear in all participant observations that we have a duty to disclose staff negligence. We will establish a system for researchers concerned about negligence to alert the PI within an established time frame and prompt notification of the relevant Freedom to Speak Up Guardian.
- *Participant engagement* (by trusts, services, units and teams who feel judged by the study sampling procedures and the study topic). We know that obtaining initial and ongoing engagement with participants is possible if done sensitively and ethically. We will reassure all trusts and services that we will not be approaching, interviewing or observing women and families directly.
- *Non-malfeasance regarding study participants.* In research information and methods, we will follow current guidance on interviewing people about potentially sensitive topics or traumatic experience[30,31] and specialist (one-to-one) advice from our PAG.

For peer review only

- *Non-malfeasance regarding PPIE representatives* (study co-applicants; PAG members; forum participants). We have structured PPIE representation across the study, so that leadership and support roles are clearly defined. We have ensured that all PPIE co-applicants and PAG members are highly experienced in research and engaging with diverse interest groups. We have also ensured linkage between the PPIE PAG lead and the study PAG lead to ensure consistency of support and representation.

Issues of anonymity, confidentiality and informed consent will be addressed in the recruitment of all participants, data collection processes and data storage. The principles of beneficence and non-malfeasance will be adhered to.

Throughout the study, the MRC/HRA guidance 'GDPR and Data Protection Act 2018: key facts for researchers' has been followed.

Study Strengths and Limitations

- This is the first study to investigate the implementation of interventions for disclosure and discussion of harm in NHS maternity care.
- Project methodology includes multi-disciplinary and user representative co-design of study questions, research focus, study outputs and dissemination strategy, with wider applicability in health service research.
- A realist approach identifies 'key ingredients for potential success' for maternity providers engaged in disclosure improvement.
- The study does not examine reasons for disinterest or disengagement among some maternity providers.

ACKNOWLEDGEMENTS

FUNDING STATEMENT
This work is supported by the National Institute of Health Research [NIHR], Health Service and Delivery Stream, grant number HS&DR 17/99/85. The study has been adopted by the NIHR Applied Research Collaboration, South London.

AUTHOR CONTRIBUTIONS
MA drafted the protocol with co-ordinated ongoing contributions from JS, RI, AH, MT, MB and CB. JH assisted with the proofreading the protocol for submission for publication.

COMPETING INTERESTS
The authors declare that they have no competing interests. The views and opinions expressed therein are those of the authors and do not necessarily reflect those of the HS&DR program, NIHR, NHS or Department of Health.

For peer review only

Bibliography

1. Berlinger N, Wu AW. Subtracting insult from injury: addressing cultural expectations in the disclosure of medical error. Journal of Medical Ethics. 2005;31(2):106-8.
2. Berlinger N. After harm: medical error and the ethics of forgiveness: Johns Hopkins University Press Baltimore, MD; 2005.
3. Wu AW, McCay L, Levinson W, Iedema R, Wallace G, Boyle DJ, et al. Disclosing adverse events to patients: international norms and trends. Journal of patient safety. 2017;13(1):43-9.
4. Vincent C, Amalberti R. Safer healthcare: strategies for the real world: Springer Nature; 2016.
5. Piper D, Iedema R. Literature Review: Incident disclosure policy, legal reform and research since 2008. 2011.
6. Iedema R, Allen S, Britton K, Piper D, Baker A, Grbich C, et al. Patients' and family members' views on how clinicians enact and how they should enact incident disclosure: the "100 patient stories" qualitative study. Bmj. 2011;343.
7. Care PSAfHaS. What is the Professional Duty of Candour? [Available from: https://www.professionalstandards.org.uk/what-we-do/improving-regulation/find-research/duty-of-candour.
8. Blythe H. Regulating the duty of candour: a report by Action against Medical Accidents on CQC inspection reports and regulation of the duty of candour. AvMA2016). www. avma. org. uk; 2016.
9. Birks Y, Harrison R, Bosanquet K, Hall J, Harden M, Entwistle V, et al. An exploration of the implementation of open disclosure of adverse events in the UK: a scoping review and qualitative exploration. Health Services and Delivery Research. 2014.
10. Agency NPS. Being open: communicating patient safety incidents with patients, their families and carers: National Patient Safety Agency; 2009.
11. Commission CQ. Regulation 20: Duty of candour. Information for all providers: NHS bodies, adult social care, primary medical and dental care, and independent healthcare Newcastle, UK: Care Quality Commission. 2015.
12. Cumberledge J. National Maternity Review: Better Births. London: NHS. 2016.
13. Health Do. Safer Maternity Care. The National Maternity Safety Strategy - Progress and Next Steps. 2017.
14. Resolution N. The Early Notification scheme progress report: collaboration and improved experience for families. London: NHS Resolution. 2019.
15. Wong G, Greenhalgh T, Westhorp G, Buckingham J, Pawson R. RAMESES publication standards: realist syntheses. BMC medicine. 2013;11(1):1-14.
16. Pawson R. Evidence-based policy: a realist perspective: sage; 2006.
17. Pawson R, Greenhalgh T, Harvey G, Walshe K. Realist synthesis: an introduction Manchester: ESRC Research Methods Programme. University of Manchester. 2004.
18. Pawson R, Manzano-Santaella A. A realist diagnostic workshop. Evaluation. 2012;18(2):176-91.
19. Project R. Quality standards for realist synthesis (for researchers and peer-reviewers). 2014.
20. Wong G. Data gathering in realist reviews: looking for needles in haystacks. Doing realist research London: SAGE. 2018:131-45.
21. Manzano A. The craft of interviewing in realist evaluation. Evaluation. 2016;22(3):342-60.
22. Mendizabal E. The Alignment, Interest, and Influence Matrix (AIIM). London: ODI. 2010.
23. Leslie M, Paradis E, Gropper MA, Reeves S, Kitto S. Applying ethnography to the study of context in healthcare quality and safety. BMJ quality & safety. 2014;23(2):99-105.

For peer review only

24. Dixon-Woods M, Leslie M, Tarrant C, Bion J. Explaining Matching Michigan: an ethnographic study of a patient safety program. Implementation science. 2013;8(1):1-13.
25. Lawton R, Taylor N, Clay-Williams R, Braithwaite J. Positive deviance: a different approach to achieving patient safety. BMJ quality & safety. 2014;23(11):880-3.
26. Denzin NK, Lincoln YS. Strategies of qualitative inquiry: Sage; 2008.
27. Phillips R. Knowledge for action: A guide to overcoming barriers to organisational change: Chris Argyris, Jossey-Bass Publishers (1993), 325 pp.,£ 23.95. Pergamon; 1994.
28. Wong G, Westhorp G, Manzano A, Greenhalgh J, Jagosh J, Greenhalgh T. RAMESES II reporting standards for realist evaluations. BMC medicine. 2016;14(1):1-18.
29. Hinton T, Gannaway D, Berry B, Moore K. The D-cubed guide: Planning for effective dissemination. 2011.
30. Elmir R, Schmied V, Jackson D, Wilkes L. Interviewing people about potentially sensitive topics. Nurse researcher. 2011;19(1).
31. Razzaghi A, Afshar L. Ethical Issues Related to Data Collection Methods in Trauma Researches. Med Ethics J. 2018;11(42):7-10.
32. NIHR. National Standards for Public Involvement in Research 2018 [http://www.donorhealth-btru.nihr.ac.uk/wp-content/uploads/2018/10/NIHR-Public-Involvement-Standards-.pdf]. Available from: https://sites.google.com/nihr.ac.uk/pi-standards/standards.
33. O'Brien BC, Harris IB, Beckman TJ, Reed DA, Cook DA. Standards for reporting qualitative research: a synthesis of recommendations. Academic Medicine. 2014;89(9):1245-51.

For peer review only

Study Flow Diagram: Strengthening Disclosure in Maternity

- PURPOSIVE SAMPLING AND APPROACH FOR CASE STUDY SITES

HS&DR Study Number:17/99/85. **IRAS ID: 262197**

STUDY PHASE 1: REVIEW AND INTERVIEW STUDY (MONTHS 1-17)

- COMPLETION OF HRA AND IRAS APPLICATIONS (MONTH 14 ON)
- RECRIITMENT OF RESEARCH ASSOCIATE (MONTH 6)
- CONTRACTS FINALISED (MONTH 9)
- LITERATURE REVIEW; DATA SUMMARIES; ESTABLISHING MAIN HYSPOTHESES
- SAMPLING FOR INTERVIEWEES AND APPROACH
- 24 SENIOR STAKEHOLDER INTERVIEWS
- REALIST ANALYSIS OF INTERVIEW AND REVIEW FINDINGS;
- WRITE UP FOR CIRCULATION AND PUBLICATION SUBMISSION

- PURPOSIVE SELECTION (3 CASE STUDY SITES) & INITIAL SITE VISITS

STUDY PHASE 2: ETHNOGRAPHIC CASE-STUDIES

Study Site 1	Study Site 2	Study Site 3
Secondary and Tertiary Services	Secondary and Tertiary Services	Secondary and Tertiary Services
10 x 3 days data collection (observations; interviews and document analysis);	10 x 3 days data collection (observations; interviews and document analysis);	10 x 3 days data collection (observations; interviews and document analysis);
Staff n<12	Staff n<12	Staff n<12
Women & families >3	Women & families >3	Women & families >3
Local Forums (n=1-2)	Local Forums (n=1-2)	Local Forums (n=1-2)

STUDT PHASE 3: STUDY WRITE-UP; OUTPUTS

Formative Data Analysis; Integrate Key Findings Study Phases 1 & 2; begin final report

SUMMATIVE FORUM FOR ALL STUDY SITES AND SENIOR STAKEHOLDERS

COMPLETE DATA ANLYSIS; BEGIN DESIGN OF STUDY OUTPUTS (FILMS AND WRITTEN OUTPUTS); DISEMINATION EVENTS; FAINL REPORT DRAFTING AND SUBMISSION

Appendix 2:

Study Recruitment And Data Management For Study Phase 1b

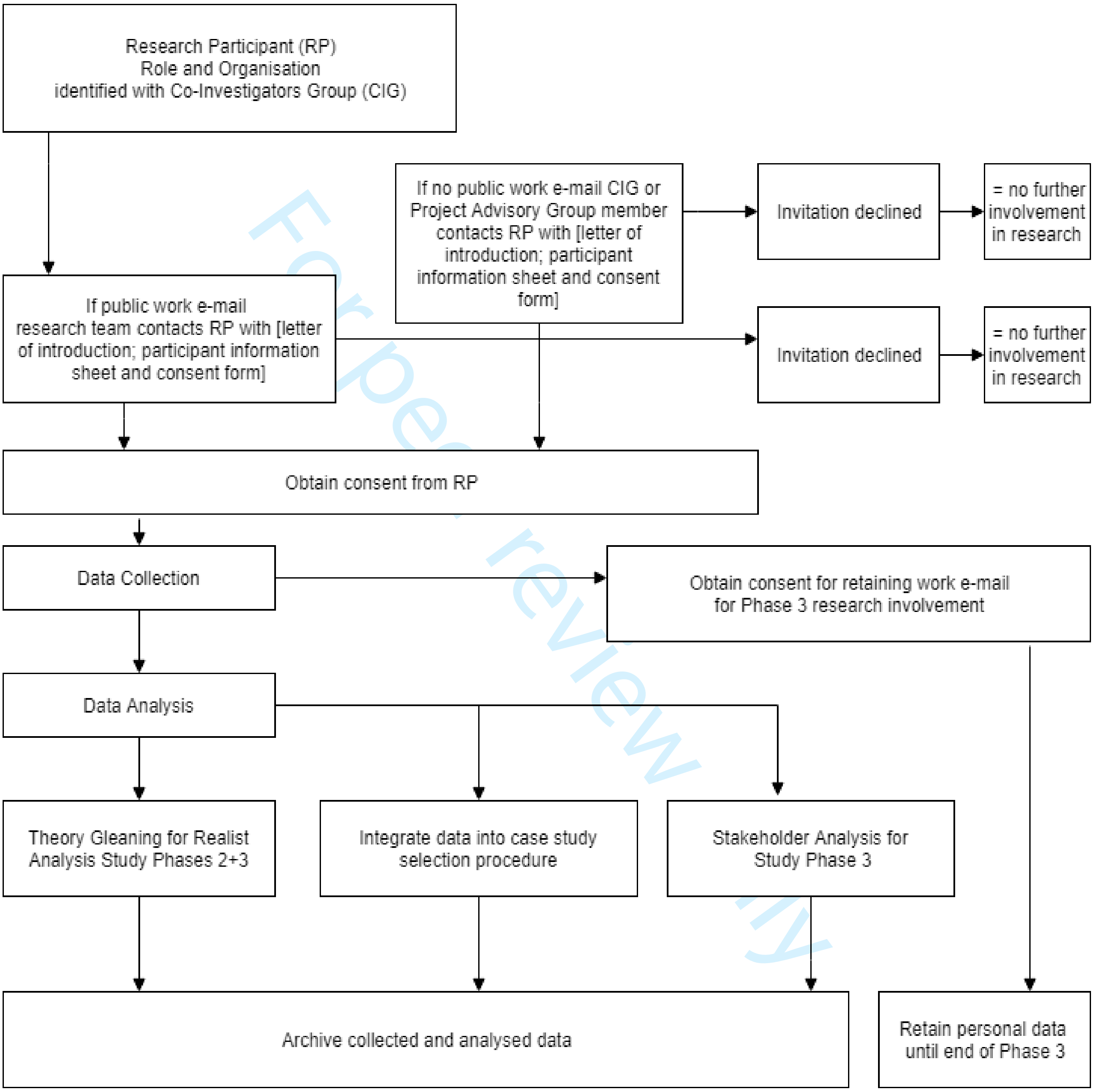

APPENDIX 3: STRATEGY FOR SHORT-LISTING POTENTIAL CASE STUDY SITES

4 potential study sites have been identified using a staged sampling frame to identify a purposive sample of organisations or services that are undertaking improvement work in OD in NHS maternity care. We have considered maternity networks, trusts or service areas or services as potential study sites. The staged sampling frame has been two-staged:

Stage 1:

Long listing potential study sites from public data sets (notably trust-level scores on 'Transparency: Learning from Mistakes League' and the NHS staff survey). This data is reported annually and shows variations between all NHS acute trusts (from outstanding levels of openness and transparency [level 1] to poor reporting culture [level 4]. This data set is established from selected metrics from the annual NHS staff survey and from the National Reporting and Learning System. Service-level scores from the annual NHS staff survey, '*Reporting on 'Errors and Incidents', selected items* [KF 29; 30;31] (see Box 1).

Box 1: 'Errors and Incidents': items from the annual NHS staff survey (by trust and location)

**Survey Item**	**Error and Incidents: by Trust and by Trust Location (Women's Health)**
KF 29	staff reported errors, near misses or incidents witnessed in last month (percentage score)
KF 30	staff reported fairness and effectiveness of procedures for reporting errors, near misses and incidents (scale summary score)
KF 31	staff reported confidence and security in reporting unsafe clinical practice (scale summary score)

These key findings are benchmarked against others trusts and for different locations [or divisions] within that trust. Women's Health, Gynaecology and Obstetrics, as well as Midwifery are all locations. Therefore, we have been able to establish a comparative picture of how staff experience incident investigation, across and between our listed sites in these areas and listed acute trusts.

We have long-listed those trust and service areas where there is good or improving performance reported in fields that indicate candour (for example, transparency, incident reporting, staff reported organisational or service responsiveness of incident reporting).

Stage 2:

Short-listing long-listed sites using combined soft intelligence from national and regional stakeholders (at Phase 1 scoping study).

Due to the sensitivity of the research topic for frontline staff as well as for services and trusts, and the anticipated challenges of organisational engagement in some trusts, services and networks, our sampling frame follows Stake's (2013) principles to:

i. maximise chances of ongoing engagement of participants in the research
ii. provide diversity of cases across contexts
iii. ensure that all cases provide good opportunities to learn about complexity and context

For peer review only

Therefore, we shortlisted only those services, trusts and networks identified as potentially available as case study sites by stakeholder or PAG recommendation.

Reference:

Stake R. E. 2013. *Multiple Case Study Analysis*. Guildford Press.

For peer review only

Appendix 4: Recruitment strategy for organisations and Individuals.

Sample Identification for Study Phase 2

Study Phase 2 (up to 4 case studies, with each case study comprising focused (group or individual staff) non-participant observations (n=25 hours); in-depth interviews with NHS staff and patients (approximately n=12); and collection and analysis of redacted documentary data (n=20 hours). Some return interviews may be arranged with staff who lead the OD improvement work (n=approximately 3 per site).

Recruitment of case study sites into the study will involve a range of approaches in order to maximise opportunities of team, service, trust and/or network engagement. These are:

a. <u>sampling strategy for the potential case study sites</u>:
The identification of any site for potential recruitment will be on the basis of their already being active in maternity quality and safety improvement work and on the basis of their likely interest in engaging with the study team (as identified by Phase 1 interviewees and our independent project advisors). We will therefore ensure that prior to our sampling for diversity or context, we have sampled to maximise our chances of ongoing engagement of services, trusts and/or networks in the study. In addition, we will identify potential sites by internet searches. These searches will assist in our closer identification of maternity safety and learning 'candour' collaborative or working groups (for example, as developed in the Health Innovation Networks or as supported by local NHS staff or parent/maternity support groups).

b. <u>use of a recommended framework for enhancing study participant recruitment:</u>
We understand that the recruitment of organisations and networks, as well as of those who work within them, is a multi-layered process. We will draw on McDonald's (2011) adapted business-model approach to recruitment to inform our recruitment plan.

We will undertake:

i. **Early and widespread familiarisation of the research project (the study question, along with the ongoing promotion of study value, legitimacy and potential benefits) across NHS maternity services.** We will initiate promotion of the study (and of the senior researchers involved) from study start date. This promotion will be established and sustained through social media (Twitter; a project blog and, on the advice of our independent project advisors, a study update newsletter) until project completion. Prior to Phase 1, and after the study has been contracted, our study team and independent project advisors will be encouraged to 'spread the word' on the research through regional and national networks. This familiarisation and ongoing promotion will be underpinned by a simple message of 'what the study stands for' and how it can be recognised.

ii. **Early identification of potential case study sites.** We will be anticipating potential sites throughout the Phase 1 research. This will include establishing short 'case study' profiles to aid us with sampling following Stake's (2013) case study sampling principles as well as by making early contact with potential sites through gatekeepers identifiable on public websites. These initial contracts (and a response after one email contact and one email reminder) will aid our assessment of their interest in potential participation.

iii. **Anticipation of multiple dimensions of recruitment**. We are aware that access to services, trusts and/or networks will require successful engagement with multiple sponsors and gatekeepers, both formally and informally. We will use tailored

messages to engage different interest groups (for example, the potential benefits of involvement in the study for patient representatives will differ from those for a group of junior clinicians or managers). We will also identify the range of influential sponsors, champions and change agents within potential case study sites to enlist their ongoing support.

iv. **Assurance to potential participants (organisations and individuals) of the scope and limits of the research across and within case study sites.** Macdonald (2011) highlights the necessity of clarifying the expectations of organisational participation and of the scope and limits of the research to be undertaken to all gatekeepers. This will be particularly important for the case-studies as participants or gatekeepers will be concerned that they may have less control over information shared through survey or interview methods. We will reiterate in all communication materials, that our non-participant observations are focused on the contexts and routines that make OD possible and that we will not be expecting to observe actual OD meetings with women or families (see case-study methods described below). We will not be recruiting women or families from the case study sites as study participants.

v. **Emphasis on the actual and potential benefits of research participation to key gatekeepers as well as to individual study participants**. We are assuming that there will be some reluctance of some potential participants to engage with this study topic. However, we have sought to enhance to benefits to participants by our study design (Phase 3 offers opportunities for networking, social interaction and sharing experiences both within the sites, from our Women and Family Forums and from our Summative Interpretive Forum). Our communications will highlight these opportunities.

vi. **The engagement of staff as potential and actual research participants will be facilitated by short presentations of the study and key study messages by the researchers at local unit meetings in different service areas**. By identified quality and safety improvement champions and by snowballing through clinical or project teams. The ongoing development of ongoing research relationships, with two named and experienced health service ethnographers, will further support recruitment processes.

For peer review only

ASSESSMENT OF 'DISCERN' PROTOCOL USING RAMESES II REPORTING STANDARDS FOR REALIST EVALUATIONS

Wong, G., Westhorp, G., Manzano, A. *et al.* RAMESES II reporting standards for realist evaluations. *BMC Med* **14,** 96 (2016). https://doi.org/10.1186/s12916-016-0643-1

	TITLE	Reported in Document	REPORTED IN PROTOCOL AS TO BE UNDERTAKEN (Y/N)	PAGES IN DOCUMENT
1	TITLE	In the title, identify the document as a realist evaluation	Y	page 1
2	ABSTRACT	Journal articles will usually require an abstract, while reports and other forms of publication will usually benefit from a short summary. The abstract or summary should include brief details on: the policy, programme or initiative under evaluation; programme setting; purpose of the evaluation; evaluation question(s) and/or objective(s); evaluation strategy; data collection, documentation and analysis methods; key findings and conclusions<br>Where journals require it and the nature of the study is appropriate, brief details of respondents to the evaluation and recruitment and sampling processes may also be included<br>Sufficient detail should be provided to identify that a realist approach was used and that realist programme theory was developed and/or refined	Y	page 1
	INTRODUCTION			
3	Rationale of Evaluation	Explain the purpose of the evaluation and the implications for its focus and design	Y	page 2 (parag.4)
4	Programme Theory	Describe the initial programme theory (or theories) that underpin the programme, policy or initiative	Y	page 2 (parag.6)<br>page 3 (parags. 2 and 3)<br>pages 5-6 (Section on

				data Analysis)
5.	Evaluation Questions, Objectives and Focus	State the evaluation question(s) and specify the objectives for the evaluation. Describe whether and how the programme theory was used to define the scope and focus of the evaluation	Y	page 3 (parag. 3).<br><br>and described in Page 3 (parag.3)
6	Ethical Approval	State whether the realist evaluation required and has gained ethical approval from the relevant authorities, providing details as appropriate. If ethical approval was deemed unnecessary, explain why	Y	Abstract
	METHODS			
7	Rationale for Using a Realist Evaluation	Explain why a realist evaluation approach was chosen and (if relevant) adapted	Y	Page 3 (parag.1)
8.	Environment Surrounding the Evaluation	Describe the environment in which the evaluation took place	Y	Page 2 (parags.1-2)
9	Describe the programme policy, initiative or product evaluated	Provide relevant details on the programme, policy or initiative evaluated	Y	Page 2 (parag.3)
10	Describe and justify the evaluation design	A description and justification of the evaluation design (i.e. the account of what was planned, done and why) should be included, at least in summary form or as an appendix, in the document which presents the main findings. If this is not done, the omission should be justified and a reference or link to the evaluation design given. It may also be useful to publish or make freely available (e.g. online on a website) any original evaluation design document or protocol, where they exist	Y	Page 2 -3<br><br>Study Design Section
11	Data Collection Methods	Describe and justify the data collection methods – which ones were used, why and how they fed into developing, supporting, refuting or refining programme theory<br>Provide details of the steps taken to enhance the	Y	Page 3-5

		trustworthiness of data collection and documentation		
12	Recruitment methods and sampling strategy	Describe how respondents to the evaluation were recruited or engaged and how the sample contributed to the development, support, refutation or refinement of programme theory	Y	PAGES 3-5<br>METHODS FOR EACH STUDY PHASE
13	Data analysis	Describe in detail how data were analysed. This section should include information on the constructs that were identified, the process of analysis, how the programme theory was further developed, supported, refuted and refined, and (where relevant) how analysis changed as the evaluation unfolded	Y	PAGES 5-6<br>DATA ANALYSIS SECTION
	RESULTS			
14	Details of participants	Report (if applicable) who took part in the evaluation, the details of the data they provided and how the data was used to develop, support, refute or refine programme theory	N/A	
15	Main Findings	Report (if applicable) who took part in the evaluation, the details of the data they provided and how the data was used to develop, support, refute or refine programme theory	N/A	
	DISCUSSION			
16	Summary of findings	Summarise the main findings with attention to the evaluation questions, purpose of the evaluation, programme theory and intended audience	N/A	
17	Strengths, limitations and future directions	Discuss both the strengths of the evaluation and its limitations. These should include (but need not be limited to): (1) consideration of all the steps in the evaluation processes; and (2) comment on the adequacy, trustworthiness and value of the explanatory insights which emerged<br>In many evaluations, there will be an expectation to provide guidance on future directions for the programme, policy or initiative, its implementation and/or design. The particular implications arising from the realist nature of the findings should be reflected in these discussions	N/A	

18	Comparison with existing literature	Where appropriate, compare and contrast the evaluation's findings with the existing literature on similar programmes, policies or initiatives	N/A	
19	Conclusions and recommendations	List the main conclusions that are justified by the analyses of the data. If appropriate, offer recommendations consistent with a realist approach	N/A	
20	Funding and conflicts of interest	State the funding source (if any) for the evaluation, the role played by the funder (if any) and any conflicts of interests of the evaluators	Y	PAGE 8

For peer review only
